# Observations on Dr. Harrison's Bill

**Published:** 1811-08

**Authors:** 


### *To the Editors of the Medical and Physical Journal.*

GENTLEMEN,

I AM concerned to find, by the perusal of your last Number, that my former observations have produced little change on the language and sentiments of your correspondent A. H. He remains equally hostile to the Medical Bill, and, as I think, is highly and unjustly prejudiced and intemperate. In order, however, that your readers may be enabled to judge and decide between us upon a question of such importance to the whole profession, I shall be glad to see a corrected sketch of the Bill published in your Journal, together with the clauses, which I repeatedly stated to have been omitted, from motives of prudence, in the present sketch, through *the recommendation* of the Members, who *had engaged to introduce, and support the Bill in Parliament.*

A. H. has, it is true, retracted two material objections, on which he formerly laid great stress, and to which I now desire to call the particular attention of your numerous readers. He asserted, that the Bill would subject the faculty to *a pecuniary payment.* He now admits, on further consideration, that it will not *so* affect the present Establishment. This is an important concession, and will, I trust, make a due impression on the minds of the regular Profession. To them I desire to be understood as asserting, that the Bill *will in no way be productive of expence or inconvenience in practice. They will be left as much at liberty to exercise their skill and talents under the projected Act as at this moment.* No practical restrictions of any kind were ever intended to be laid laid upon them. Such has been the uniform language of the Reformers, as set forth in their different statements, and I defy the most prejudiced objector to convict them of harbouring other views. Should any one remain sceptical on this point, let him examine the various printed documents. He will soon find conviction, and will, I hope, like my present opponent, have the candour to acknowledge it.

2dly. Your Correspondent entertained an opinion, that the Magistrates would be empowered under the Act to licence quacks in all succeeding generations. He now finds the Magistrates will be only authorized to interdict or sanction the practices of the present race of Quacks. As these die away or retire they cannot supply the list with new adventurers. Their discretion being wholly restricted to the present race, empirical practice must necessarily terminate with this generation. The Reformers, well convinced of the mischievous proceedings of empirical pretenders, were desirous to put an immediate stop to their interference, and have only relinquished the object from prudential considerations. Convinced as they have long been of the folly of such an attempt, they have confined their exertions to a slower, but they trust more certain process. In order to comprehend the policy and efficacy of the plan, let us suppose the empirical establishment of the United Kingdom to consist of 100,000, and I am disposed to believe, that including village doctors and midwifes, the calculation is not over rated. Should only nine in ten be suppressed at the first meeting of the Magistrates, the register will be limited to 10,000, consequently, the destructive career of 90,000 will be for ever arrested. And will humanity gain no relief from such a diminution? Will medical practice derive no benefit from the suppression? Or shall we say with Haman, "all this availeth nothing so long as we see Mordecai the Jew sitting at the King's gate."

It seems from this estimate that 10,000 will still remain to prey upon the lives of deluded invalids, and curtail the profits of regular men. And let us see what will be likely to happen to them. They are now for the most part general dealers and universal practitioners, whereas most of them would have their future proceedings limited by the Magistrates to particular cases. The persons, for example, licensed to set and replace bones would not be suffered with impunity to tease joints suffering from scrofulous inflammation, from rheumatism, or other pains. When thus confined to particular lines of employment, they would find the field too small for profitable culture, and many of them would voluntarily abandon the curative art for better speculations. In this way and by the controul of the Bench of Magistrates, *who can at pleasure*

*withdraw*

*withdraw the licences*, the practice of many empiricks would thus be arrested long before the termination of their natural lives. A Bill calculated to effect these objects, so desirable to medical science, and so beneficial to incautious invalids, should not, I think, be hastily condemned and abandoned, except from the pleasing prospect of introducing another of greater vigor and more immediate operation. Of the fate of such a Bill, in its progress through Parliament, no person acquainted with the prejudices of the higher orders can entertain a doubt. We are consequently reduced to the alternative of leaving quackery to continue its destructive ravages *unmolested*, or of opposing it by a cautious interference. The recent failure of Lord Sidmouth's Bill to restrain the religious effervescence of illiterate and ignorant preachers, and the checks imposed upon Sir S. Romilie's benevolent attempts to proportion the punishment of criminals to the enormity of their offences, ought to impress us with a becoming diffidence of our own success in Parliament, *unless we adopt moderate measures and proceed in the most cautious manner.*

But it seems "no man in his senses would think of appealing to their Worships the Justices at the general or petty Sessions, as a competent tribunal to decide on the practice of physic?" From the opinion I entertain, I have always placed the Magistrates of Britain, acting as they do without fee or reward, among the most useful and deserving members of the Empire. In their official situations they have certainly the best opportunities of becoming acquainted with and deciding upon the general characters of empirics. But if the Bench of Justices are incompetent, in whom shall we repose confidence? Surely not in our noble selves, interested as we must be in the decision. Since it is a maxim in Jurisprudence, that no person shall sit as Judge when he is liable to be prejudiced or influenced by selfish considerations.

3dly.. The great difficulty in the way of reform arises, as I conceive, from the Corporate bodies themselves. The members have contrived by private regulations and selfish by-laws to acquire a superiority in practice, and in professional character, over the Licentiates; the commonalty of Surgeons, and independent practitioners. These gentlemen, equal in talents and learning to their favored rivals, are forced to move in a lower sphere, merely because they are not suffered to contend for the highest rank in their respective colleges. These mortifications produced in direct violation of the acts of parliament and charters founded on them, are intended to serve the private views of the parties in possession. Hence the chief obstacle to reform. The persons in power satisfied with their condition meditate no change, and oppose oppose every measure, however mild and necessary, because they are afraid of some concealed purpose, some hostile motives. While the oppressed Faculty appear to be resolved to discourage every scheme of correction, which does not promise to loosen their fetters and remove their disabilities. The exaltation of medical men, the improvement of medical science, and the fall of quacks, are esteemed subordinate considerations with the respective parties. Feelings of this kind afford, as I apprehend, the true grounds of opposition to the proposed Bill. It is viewed with distrust by the Fellows of the College of Physicians, and Governors of the College of Surgeons, while it is disdainfully rejected by the Licentiates and commonalty of Surgeons, because, in its present shape, it offers to them no immediate prospect of College honors, and emoluments. I think I can perceive that A. H.'s hostility originates more in disappointed expectations of a radical reform, than in settled dislike to the Bill itself. Should he be really actuated by such considerations, I do not despair of seeing him fully reconciled to the scheme, when the parts omitted by the advice of parliamentary friends shall have been again restored, as they are intended to be, to their old places in the Bill. They were removed, as I formerly observed, from a desire not to irritate the Corporate Bodies, rather than from any conviction of their impropriety or inexpediency. The necessity for a more liberal and just interpretation of the Medical Statutes and Bye-laws is generally admitted, and I deem the present times to be particularly auspicious to such an inquiry. Under the enlightened direction of the Prince Regent, I think exertions will be made to improve the condition and increase the happiness of all descriptions of people, by correcting and reforming abuses in the several departments of the State. Few have suffered longer or more grievously than medical men. It is to be hoped, that in consideration of their laborious and important duties, they will meet with speedy and effectual redress. I am ready, for my own part, to come forward with pecuniary as well as personal assistance, to effect an object of such importance to the curative art, and humanity at large. If we be really desirous to shake off an odious aristocracy, let us not consume our time in querulous complaints or bitter invectives against each other, but proceed to form an union in the British Capital of professional men of every denomination and rank. The Fellows of the College amount to about 60, the Licentiates to 300. The College of Surgeons is governed by 21 Surgeons, to the exclusion of several thousands in and near London. The Company of Apothecaries is equally limited, while the independent practitioners are very numerous. A

contention contention between the privileged Members and great body of the Faculty would, as it ought, terminate in the overthrow of monopoly, and unjust assumption, in favour of liberal views and equal privileges. Success is, I am persuaded, within the reach of the oppressed party, but in order to attain it a respectable force must be collected and organized, otherwise their remonstrances will be neglected and disregarded.

Your's, &c.
H. R.

[To be continued.]

